# The Fungal and Bacterial Interface in the Respiratory Mycobiome with a Focus on *Aspergillus* spp.

**DOI:** 10.3390/life13041017

**Published:** 2023-04-14

**Authors:** Anna Rozaliyani, Budhi Antariksa, Fariz Nurwidya, Jamal Zaini, Findra Setianingrum, Firman Hasan, Husna Nugrahapraja, Humaira Yusva, Heri Wibowo, Anom Bowolaksono, Chris Kosmidis

**Affiliations:** 1 Department of Parasitology, Faculty of Medicine, Universitas Indonesia, Jakarta 10430, Indonesia; 2 Indonesia Pulmonary Mycoses Centre, Jakarta 10430, Indonesia; 3 Department of Pulmonoloy and Respiratory Medicine, Faculty of Medicinie, Universitas Indonesia, Persahabatan National Respiratory Referral Hospital, Jakarta 13230, Indonesia; 4 Life Science and Biotechnology, Bandung Institute of Technology, Bandung 40312, Indonesia; 5 Magister Program of Biomedical Sciences, Faculty of Medicine, Universitas Indonesia, Jakarta 10430, Indonesia; 6 Department of Biology, Faculty of Mathematics and Natural Sciences (FMIPA), Universitas Indonesia, Depok 16424, Indonesia; 7 Manchester Academic Health Science Centre, Division of Infection, Immunity and Respiratory Medicine, Faculty of Biology, Medicine and Health, University of Manchester, Manchester M23 9LT, UK

**Keywords:** lung mycobiome, chronic lung diseases, immunological response, *Aspergillus* spp.

## Abstract

The heterogeneity of the lung microbiome and its alteration are prevalently seen among chronic lung diseases patients. However, studies to date have primarily focused on the bacterial microbiome in the lung rather than fungal composition, which might play an essential role in the mechanisms of several chronic lung diseases. It is now well established that *Aspergillus* spp. colonies may induce various unfavorable inflammatory responses. Furthermore, bacterial microbiomes such as *Pseudomonas aeruginosa* provide several mechanisms that inhibit or stimulate *Aspergillus* spp. life cycles. In this review, we highlighted fungal and bacterial microbiome interactions in the respiratory tract, with a focus on *Aspergillus* spp.

## 1. Introduction

Growing evidence suggests that the human lung microbiome plays an essential role in the control of the immunological response, the metabolic activity, and the development of long-term inflammatory illnesses [1,2]. Classical theory reveals that the lung was considered sterile until recently, when the use of novel culture-independent methods such as sequencing tools were invented [3,4]. The sterility of the lung has previously been the subject of vigorous scientific investigations, because healthy adults breathe in more than 7000 L of air each day, which contains microbes [3]. By using this technique, the diversity of the pulmonary microbiome has been revealed in the lung [2,5,6,7]. However, due to the sampling techniques, investigations into the microbiome in the lung have remained limited and the lower respiratory tract is less accessible.

Furthermore, a study by Gusareva et al. has revealed that fungi account for a significant proportion of microorganisms in the air, reaching 82% [8]. However, the study of the microbiome has focused more on elucidating the bacterial composition in the lung, despite identifying other microorganisms such as fungi, which may also contribute to the pathogenesis of chronic respiratory disease [2,9,10,11,12]. Various fungi might be found to be widely spread in the environment, some of which impact pulmonary health [2,12]. The role of *Aspergillus*, as well as other molds and yeast, in promoting the exacerbation of respiratory diseases has remained largely unexplored [2,12].

Comprising hundreds of species, *Aspergillus* has the potential to be more pathogenic owing to its potential for resilience and ubiquity in the environment [13,14]. The tremendous survival capability of *Aspergillus* is supported by various immune evasion strategies that allow it to adapt to hostile environments [15,16,17,18]. The co-existence of *Aspergillus* and pulmonary diseases such as asthma, cystic fibrosis (CF) and chronic obstructive pulmonary diseases (COPD) poses a high risk of worsened clinical outcomes for patients [19,20,21,22,23]. Consequently, the clinical spectrum of aspergillosis, such as chronic pulmonary aspergillosis (CPA) and allergic bronchopulmonary aspergillosis (ABPA), has been formed due, in part, to these interactions [22].

Patients with pre-existing cavities caused by tuberculosis or COPD are more likely to develop CPA [24]. Chronic pulmonary aspergillosis (CPA) has been acknowledged as a serious lung disease, likely followed by a progressive course and associated with a diverse spectrum of manifestations [25]. Moreover, aspergillosis might lead to allergic inflammatory responses due to its colonization in the respiratory tract. This condition, termed allergic bronchopulmonary aspergillosis, can be seen in patients with asthma and CF [26]. A study by Isa et al. (2021) showed that airway inflammation in asthmatic children was affected by indoor air particulates, including fungal spores. The airway inflammation that was caused by eosinophil and neutrophil activation and degranulation markers is associated with exposure to particulate matter with an aerodynamic diameter  ≤ 2.5 μm (PM_2.5_), nitrogen dioxide (NO_2)_, *Trichosporon asahii*, *Papiliotrema bandonii*, and *Aspergillus clavatus*. Furthermore, it was shown that there is a correlation between the asthma phenotype and indoor pollutants, in which a fungi profile can be identified [27,28,29].

Fungi are commensal organisms, interacting with bacteria and the host to ensure a healthy microbiome. Though modest in number, fungi have enormous genomes and may operate as keystone species in the microbiome [30]. The mycobiome is the fungal biota of the human microbiome. Members of the mycobiome possess the ability to switch from commensalism to pathogenicity, which is frequently dependent on the presence of colonizing microbial taxa. The disruption of commensal populations might have an impact on both local and peripheral immune responses, as well as potentially exacerbating disease states [31,32]. Recent research has indicated that airway microbial populations might contribute to preserving airway health [33,34]. This review provides an overview of fungal and bacterial interaction in the respiratory tract, with a focus on *Aspergillus* spp.

## 2. Lung and Lower Respiratory Tract Microbiome

### 2.1. Bacterial Microbiome

Culture-independent techniques using the 16S rRNA gene found that Firmicutes, Proteobacteria, Bacteroidetes, and Actinobacteria occupied healthy lungs [35]. Intriguingly, the microbiota of the lungs contains an astonishing 2000 bacterial genomes per cm^2^ [36,37,38]. The lung microbiome is predominantly formed through the aspiration of the oropharyngeal secretion or direct contact with its mucosa. Therefore, there may be an increased possibility of direct interaction between the microbiota of the upper and lower airways [7,39].

According to a previous study, the extent of variance from neutrality is linked to the severity of lung disease [40]. The neutrality idea holds that all microorganisms have an equal probability of acquiring and thriving in a particular habitat, but they also risk dying there. This paradigm revealed several niches of interaction, including microorganism colonies which are determined by ambient resources or inter-species relationships [40]. The diversity of microbiota in the lung is affected by lung diseases. The loss of species richness in the lung microbiota due to bacterial overgrowth is linked to the progression of diseases such as cystic fibrosis [41,42].

There is a continuous flux of microorganisms renewing and replacing the microbial ecosystem. Most of them are classified into Bacteroidetes and Firmicutes, which dominate the human lung environment [35]. Meanwhile, in mice Proteobacteria and Actinobacteria are predominate [43,44,45,46,47,48,49]. The human lung microbiome depicts larger spatial variation depending on the site relative to the alveoli [44]. The analysis of low-density populations might be complicated, as bias is often relatively apparent due to DNA contaminants that predominate over the authentic DNA sample. Using extraction procedures at densities of less than 106 bacteria per mL of the material may significantly affect the composition and quantity of the identified bacteria [50].

It has been difficult to define a “typical” microbiome that maintains a state of homeostasis between microbiota and the host cells owing to their heterogeneity among individuals. A lack of evidence also exists as to whether the microbiome profile can indicate or contribute to good lung health. Moreover, symbiotic species such as *Faecalibacterium prausnitzii* have been identified as likely favorable lung bacteria [51]. It has been found that most lung microbiota might be governed by dysbiosis in lung disorders such as asthma and COPD [35,36]. The precise role and causative relevance of this dysbiosis in the development and progression of asthma are still unknown. The phylum classification of bronchoalveolar lavage from severe asthmatic children differed from control subjects [36]. The most abundant phylum was Proteobacteria, followed by *Firmicutes* (primarily *Streptococcus*), *Bacteroidetes* (mainly *Prevotella*), and *Actinobacteria* in decreasing order [36]. *Staphylococcus* and *Haemophilus* were more prevalent in asthmatic people, while *Prevotella* was frequent in controls [36].

### 2.2. Fungal Microbiome (Mycobiome)

In contrast to the bacterial microbiome, which has been investigated extensively, there has been little discussion about the mycobiome because of several difficulties regarding this field of study. First of all, fungal databases are limited, insufficient, and inaccurate, including fungal taxonomic, genomes, and ribosomal databases [12,30]. Many fungi evade identification by conventional culture procedures, and there is still a technological barrier to molecular based-methods [30,52]. Furthermore, combination steps for nucleic acid isolation from fungal cells are often needed, including enzymatic, chemical, and mechanical lysis, making this process more challenging [12]. The sequencing primer choice also affects fungal taxa differentiation [12]. Researchers also encounter difficulties in studying the airway microbiome [53].

The average fungal variety in the lungs is smaller than that of bacteria [3], but it has a more significant coefficient of variation, which is measured by the ratio of standard deviation to the average when compared to similar samples of bacteria [54]. Most of the fungi identified in the human respiratory tract belong to the phyla *Basidiomycota* and *Ascomycota*. The most common species of fungi found in lung tissue included *Cladosporium*, *Eurotium*, and *Aspergillus* [55].

**Figure 1:**
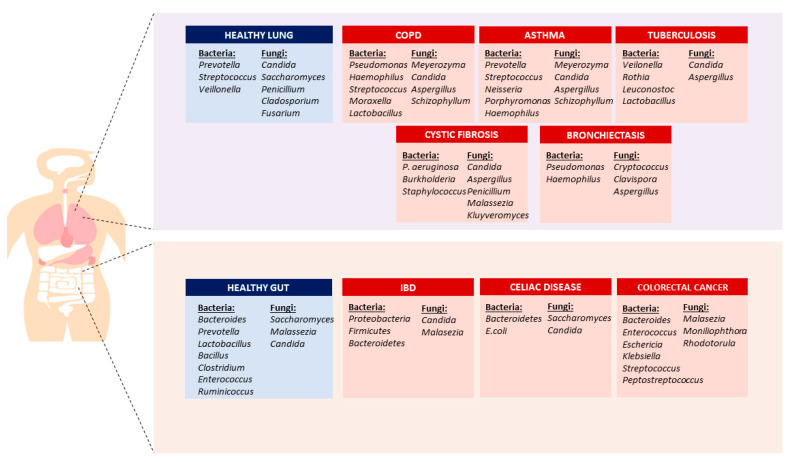
Human microbiota composition in the gut and lung. Different bacterial and fungal microbiota were observed in healthy and diseases conditions, such as chronic obstructive pulmonary diseases, asthma, tuberculosis, cystic fibrosis, and bronchiectasis, diseases in the lung, and inflammatory bowel disease, celiac disease, and colorectal cancer in the gut [7,36,37,55,59,65,66,67,68,69,70,71,72,73,74,75,76,77,78,79,80,81,82,83].

There is a huge variation in the fungal species found in the respiratory tract within the individual (Figure 1). Moreover, the fungal communities of various patients with the same disease have even been found to be distinctive [56]. Preliminary work on the microbiome in sputum revealed that Ascomycota predominated the fungal microbiome in both COPD patients and healthy controls [57]. Additionally, another study investigated the fungi living in the airways of asthmatic and healthy subjects and showed that the most abundant ones were *A. fumigatus* and *C. albicans* [58]. The airway mycobiota was a diverse population with a significant degree of individual variation. Significant alterations in the fungus found in the lung were related to the severity and longevity of asthma and inflammatory markers [27,58].

The correlation between *Aspergillus* and bronchiectasis were observed in several conditions, such as in refractory asthma patients [20,59,60]. Patients with *Aspergillus*-dominant mycobiomes have a higher frequency of bronchiectasis exacerbations, proven by qPCR, which is related to airway *Aspergillus terreus* [59,60]. Each mycobiome profile is linked to distinct *Aspergillus*-related illness states, along with “immunoallertypes” of sensitization and its links to clinical outcomes [59]. In comparison with a house-dust mite chemokine-dominant group, a fungal-driven pro-inflammatory group is linked with poor outcomes, including low lung function and worsening disease severity [59,60].

Inquiries into the involvement of fungi in chronic inflammatory airway illnesses are becoming increasingly popular. Several factors are associated with generating dysbiosis, which might arise from the microorganism and its milieu and the inflammation response from the host [53]. Respiratory tract dysbiosis promotes an alteration in the immunological response, which affects the development environment for microorganisms in the airways. Variations in the composition of the baseline respiratory tract microbiome can elucidate the so-called frequent-exacerbator phenotype seen in some disease states [61].

The lung mycobiome may exert significant inflammatory effects, contributing to or exacerbating lung diseases. Fungi possess pathogen-associated molecular patterns (PAMPs) comprised of mannans, chitin, and glucans in their cell walls. To activate the immune cells, which subsequently yield inflammation, the PAMPs will be recognized by the structure called pattern recognition receptors (PRRs) [62,63]. As fungi are ubiquitous in the milieu, the respiratory epithelium might be a large structure likely to be exposed to fungi. These processes then evoke adaptive and innate immune responses by activating macrophages and the T cell response associated with cytokine secretion, and activating the immune system. In a nutshell, owing to its potential to promote inflammation, the mycobiome may have a significant influence on the way the respiratory–immune system adjusts and leads to lung injury [64].

### 2.3. Bacterial–Fungal Interaction

**Figure 2:**
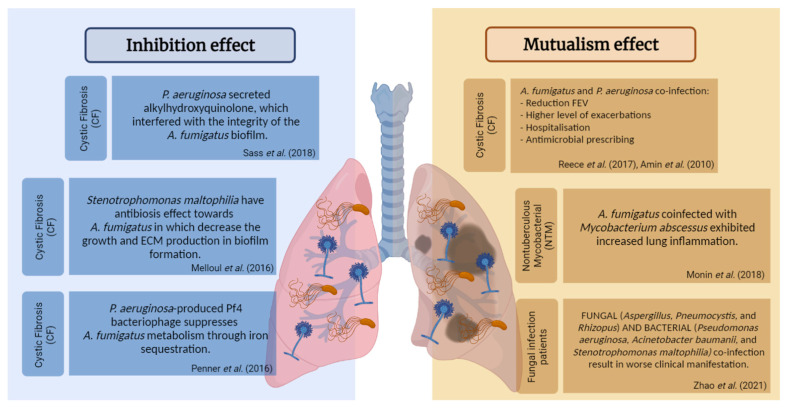
Fungal and bacterial interaction in the lung, illustrating the inhibition as a means of bacterial defense against *Aspergillus* and the mutualism as the beneficial interactions between them, resulting in the severity of the disease [101,104,109,110,111,112,113].

The respiratory tract has the most extensive surface area for bacteria and fungi to interact. Bacteria and fungi interact using quorum-sensing molecules and proteins [84,85,86,87,88]. Their dynamic interaction might affect the growth and physiological changes of bacterial and fungal colonies in the lung (see Table 1). It is also possible that polymicrobial contact involving the fungal community may alter the pathogenicity of the bacteria [89,90]. The relationship between the fungal–bacterial interaction and the host could alter the colonization by fungi, bacteria, or both [91]. A recent study revealed mycobiome identified in various types of cancer, and showed interactions with the microbiome [92]. Considerable evidence shows that many interactions between bacteria and fungi are believed to be mediated by secreted chemicals (Figure 2). Interestingly, such extracellular signaling molecules are frequently implicated in quorum sensing in single-species communities, showing that the effect of one organism on another may depend on the density of the population of the community [89,93,94,95,96,97,98]. Fewer connections between fungal and bacterial microbiota in the lung were seen in asthma patients, and this change was irreversible after inhaled corticosteroid therapy [66].

**Table 1:** Fungi and bacteria interactions in the lung.

No.	Mode of Interaction	Source of Isolates	Reference
1.	*Pseudomonas aeruginosa* inhibited *Aspergillus fumigatus* biofilms from conidia in CF isolates compared to non-CF.	CF and non-CF patients	[99]
2.	Four typical phenazines released by *Pseudomonas aeruginosa* suppressed the growth of *Aspergillus fumigatus* by inducing ROS and NOS.	Murine aspergillosis models	[100]
3.	Pyoverdine, a chemical made by *P. aeruginosa*, may be able to collect iron from the environment, which would prevent *A. fumigatus* from growing as a result of nutritional shortage.	*A. fumigatus* isolates were from ATCC (ATCC 90240), ATCC 46645 *sidA ftrA* mutant, while the *P. aeruginosa* isolates were obtained from CF patients.	[101]
4.	*P. aeruginosa* produced dirhamnolipids that promoted the secretion of dihydroxynaphthalene (DHN) and pyo-melanin from *A. fumigatus*, which surrounded their hyphae to facilitate the *P. aeruginosa* binding, leading to the inhibition of the fungi growth through the blocking of β1,3 glucan synthase (GS) activity.	Murine aspergillosis models	[102]
5.	*P. aeruginosa* secreted alkylhydroxyquinolones, which interfered with the integrity of the *A. fumigatus* biofilm.	CF patients (pediatric)	[103]
6.	*P. aeruginosa*-produced Pf4 bacteriophage suppressed the *A. fumigatus* metabolism through iron sequestration.	CF and non-CF patients	[104]
7.	*A. fumigatus* inhibited the biofilm formation of *P. aeruginosa* through gliotoxin production.	CF patients	[105]
8.	*A. fumigatus* overcame iron starvation by releasing its hydroxamate siderophores, thus promoting iron and depriving *P. aeruginosa*.	Isolates 10 AF, AF13073, AfΔsidA, AF46645, AfΔsidC, AfΔsidF, AfS77, PA14, pvdD-, pvdD-pchE-	[106]
9.	*P. aeruginosa* may encourage fungal growth by secreting volatile organic chemicals.	*Aspergillus fumigatus* CBS144-89, *Pseudomonas aeruginosa* PAO1	[107]
10.	The coexistence of *A. fumigatus* may enhance *P. aeruginosa*’s phenotypic and genetic changes, increasing bacterial virulences.	*A. fumigatus* 53470 (AF53470), *A. fumigatus* ATCC 36607 (AF36607), *P. aeruginosa* 56402 (PA56402) and *P. aeruginosa* ATCC27853 (PA27853) were used in this study.	[108]

Many mechanical interactions between bacteria and fungi have also been documented, extending from direct interaction and the aggregation of bacterial cells with fungal hyphae to the formation of biofilms between bacteria and fungi [90,114,115]. The inhibition of fungal viability has been linked to such cellular interactions which may be undertaken through the bacterial secretion of antifungal chemicals into the local milieu. The mechanisms of toxin transfer might move directly into the fungal cell via secretion pathways or nutrition restriction [116]. Another example of what is meant by bacterial–fungal interaction is the environmental modification in terms of pH change [117], which can affect *C. albicans* hyphae formation [118]. Mutually beneficial interactions are also possible in mixed-biofilm environments, where the different species shield each other against an invading immune response or antimicrobial agent [116].

Lung epithelial surface microbes may now be identified using DNA-based culture-independent approaches. The fungi from CF and COPD patients’ sputum or bronchoalveolar lavages have been studied [55,119,120,121,122,123]. There has been no detailed investigation of the interaction between *A. fumigatus* and the lung bacteria primarily associated with COPD. By contrast, the coexistence of *A. fumigatus* and *P. aeruginosa* has been explored in individuals with CF, notably in elderly patients with chronic infection [124].

In response to the presence of bacteria, fungi produce an extracellular matrix to protect themselves. Bacterial cells were always observed sticking to *A. fumigatus* hyphae based on the modelling provided in previous studies [102,109]. Evidence suggests that an extracellular component called galactosaminogalactan (GAG) from *A. fumigatus* is essential for *P. aeruginosa* adhesion, and it is produced when bacteria invade the fungus [125]. Additionally, an electron-dense substance was detected on the extracellular matrix of *P. aeruginosa*–*A. fumigatus* mixed biofilms, which were determined to be dihydroxy naphthalene (DHN)- and pyo-melanin [125]. Furthermore, thick cell wall production is definitively associated with another physiological response of fungi interaction to bacterial stress. Additionally, the hyphae become extremely ramified when bacteria are present, with short ramifications at the terminals. Dirhamnolipids and maltophilin are the chemicals released by *P. aeruginosa* and *Stenotrophomonas maltophilia*, respectively, that increase the thickness of the fungal cell wall [125,126,127,128].

The lungs of cystic fibrosis patients colonized by *A. fumigatus* and *P. aeruginosa* might be linked to a worse disease prognosis than separate infections with both pathogens [110,129]. It is also likely that *P. aeruginosa* has the potential to inhibit *A. fumigatus* growth [130,131]. *P. aeruginosa* mediates this interaction via the production of quorum-sensing molecules, and likely also its virulence factors [99,131,132]. The LasIR quorum-sensing system has been implicated in inhibiting *A. fumigatus* biofilms. In addition, phenazine derivatives pyrrolnitrin and pyocyanin have been shown to inhibit the growth of fungi [99,132].

## 3. Gut-Lung Microbiome Axis

**Figure 3:**
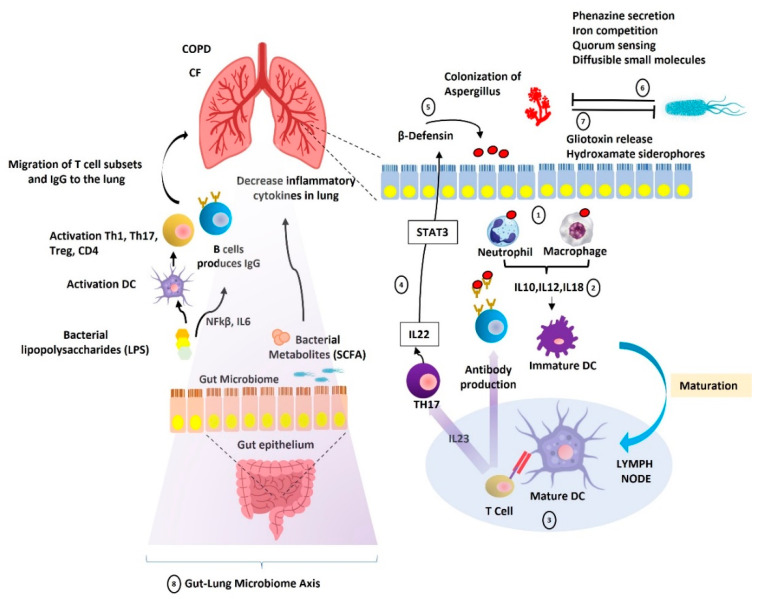
Dynamic interaction between fungal and bacterial microbiome and their immunological responses. Alveolar macrophages recognize *Aspergillus* in the lung and recruit neutrophils, macrophages, and dendritic cells (DC), which play a critical function as Natural Killer (NK) cells (1). Additionally, the innate immune response to fungi adds to the generation of specific pro-inflammatory cytokines, which promotes DC maturation in the lymph node (2). DC maturation results in an adaptive immune response, including Th17 and anti-*Aspergillus* antibodies (3). Th17-derived IL22 may upregulate the expression of the defensin protein in the human alveolar epithelium via STAT3 (4). This protein then affects the microbiome’s composition in the lungs (5). *Aspergillus* colonization is also governed by antagonistic interactions with the *P. aeruginosa* colony in the lung via various mechanisms. *Pseudomonas aeruginosa* may impede *Aspergillus* colonization by various processes, including the secretion of phenazine, iron competition, quorum sensing, and the creation of tiny diffusible compounds (6). Additionally, *Aspergillus* used multiple inhibitory strategies against *P. aeruginosa* colonies, including gliotoxin release and phenotypic modifications (7). The metabolites of the gut microbiome, such as LPS and SCFA, may alter the lung immune response and decrease lung cytokine production, respectively (8). DC: Dendritic Cells, NFkβ: nuclear factor kappa-light-chain-enhancer of activated B cells, STAT3: Signal Transducer and Activator of Transcription 3.

The gut and respiratory mucosa layer play an essential role in the mechanical barrier against microbe invasion. Their interaction with the typical microbiota results in pathogen resistance [133]. A growing body of literature recognizes the importance of how the gastrointestinal and respiratory tract communicate in terms of immune and microbial levels (Figure 3) [134,135,136,137,138]. It has now been well established from various studies that fluids, particles, and microorganisms stored in the nasal cavity of mice can be identified in the gastrointestinal tract of the mice a short time later [139].

Extensive research has demonstrated that the gut microbiota is critical in modulating local immune responses [140]. Crosstalk between the gut microbiome and the response to immune mucosal system mediated by pro-inflammatory and regulatory signals also likely promotes the extravasation of neutrophils from the bloodstream to the local immune response [141]. Moreover, the intestinal microbiota can play an essential role in addressing the issue of developing adaptive immune responses [142]. Non-pathogenic strains of Salmonella might contribute to preventing the ubiquitylation of nuclear factor-κB (NF-κB) inhibitor-α (IκBα), which was yielded to downregulate the inflammation responses in GIT epithelial cells [143].

The invasion of the respiratory system by microorganisms provides core signals for the development of local immune cells, which has significant effects on the organism [144]. Pre-clinical research depicts that microbes’ colonization of the airways as associated with the maturation and control of the airway immune cells. Germ-free mice had higher Th2-associated cytokines and IgE levels in their airways, which promotes allergic airway inflammation [145]. The presence of commensal bacteria in the lungs has been shown to inhibit Th2-associated cytokine production following an allergen challenge and to stimulate the development of regulatory cells in children [144,146]. The development of resident memory B cells in the lungs also necessitates exposure to antigens produced by the lung microbiota, which is particularly important in viral immunization against influenza [147].

Two potential mechanisms were initially characterized as the immune regulation via the gut-lung axis: activating the Toll-like receptor (TLR), and interfering T and B cell homing. However, exemplar mechanisms of how the intestinal microbiome strives to exhibit systemic immunomodulatory effects remain unexplored [148]. The interaction likely mediates the immune signaling pathways in the intestinal between the gut microbiota and a recognition receptor (TLR). This interaction subsequently promotes the downstream signaling of activating transcription factor NF-κB, essential to produce several genes that regulate innate immunity and inflammation [149]. Moreover, the intestinal microbiota also plays a critical role in maintaining TLR signaling [150,151].

Migrating lymphocytes to specific tissues is essential for acquiring an effective immune response. In a nutshell, interaction microbes with dendritic cells (DCs) in the intestine might induce the activation of different T cells inside the mesenteric lymph node (MLN), and are likely to produce several cytokines such as IL-6, TGF-β, INFγ, and IL-10. T cells are reported, subsequently, to achieve immune homing molecules such as CCR4 and CCR9, which enable T cells to migrate to non-Lymphoid tissues such as the gut, skin, and lung. Additionally, T cells also receive the ability to migrate to non-lymphoid tissues through direct interaction with mucosal DCs, leading to the enhancement of the expression of integrin α4β7 and CCR9 on T cells, thus facilitating T cell migration to the small intestine via the intestinal ligand MAdCAM-1 and the chemokine CCL25 [152]. Conversely, lung DCs favor CCR4 expression on T cells, allowing activated T cells to enter the lung through raised CCL17 levels [153]. Lung DCs have been shown to increase the presence of gut-homing integrin on T cells, driving them to the GI tract [154]. Once this has taken place, activated T cells transferred to the respiratory mucosa then activate protective and anti-inflammatory responses. Additionally, the production of essential immunomodulatory metabolites from bacteria, such as short-chain fatty acids (SCFAs), might also adjust the inflammation level, leading to the perturbation of the gut–lung axis [148].

Some studies have shown the prominent interactions between intestinal *Candida albicans* and *Aspergillus fumigatus* residing in the lung as the representation of a gut–lung mycobiome axis [155,156,157,158,159]. The overgrowth of *C. albicans* and intestinal bacteria caused by oral antibiotic therapy in mice results in the production of prostaglandin E2 (PGE2) and promotes M2 macrophage polarization in the lungs [160]. The increased concentrations of PGE2, specifically, are induced by *C. albicans* mannan. PGE2 pathways constitute an important route for Th17 expressions, and both are immensely modulated by *C. albicans* [159]. The crosstalk between *C. albicans* and *A. fumigatus* has caused the modulation of Th17 in the lung and airway inflammation responses [155]. It can be concluded that the constant inhalation of *A. fumigatus* alone is not enough to cause allergic reactions such as in ABPA [156]. Gut dysbiosis is required, and plays an important role in the pathogenesis of fungal allergic diseases in the lung [156,160].

It has been previously observed that the microbiome is associated with *Aspergillus*-related allergic airway disease. One study, by Noverr et al., examined the evidence for whether antibiotic and mycobiome interaction might induce allergic airway disease [161]. The allergic response was observed by increasing eosinophils, mast cells, interleukin 5, IgE, and mucus production in mice treated with an antibiotic. The perturbation of the GI bacterial population and the increasing yeast number were shown in the mice treated with antibiotics and, interestingly, they also induced a CD4 T-cell-mediated allergic airway in response to the exposure of *Aspergillus fumigatus* [161]. Hence, it could conceivably be hypothesized that antibiotic exposure might alter the diversity of the GI microbiome, subsequently leading to the change in respiratory immune responses to *Aspergillus*, contributing to allergy-induced obstructive pulmonary disease [161].

It is now well established due to a previous study that a population with a healthy microbiome has an essential role in maintaining balanced immunity and preventing the overgrowth of fungi [162]. The presence of commensal bacteria might suppress the level of IgE and basophil in circulation, which play a critical role in the allergic response. It is known that IgE antibodies might potentially enhance IL-3 responsiveness when they interact with bone marrow-resident basophil precursors. Subsequently, this interaction increases the number of mature basophils and aggravates the allergen-induced inflammation [162].

## 4. Discussion

### 4.1. The Fungal and Bacterial Interface in Specific Respiratory Diseases Entities

Complex interactions between fungi and bacteria in the lung are still limited and unexplored. However, previous studies which focused on several diseases are provided here.

#### 4.1.1. Chronic Obstructive Pulmonary Disease (COPD)

Impairing the innate immune system caused by repeated exacerbation in individuals with persistent COPD might lead to the perturbation of the lung microbiome with regard to quantity, variety, and composition [61,163,164,165,166,167,168]. A significant analysis of the bacterial load and airway inflammation when comparing individuals with stable and exacerbated COPD, conducted by Singh et al., demonstrated that exacerbations of COPD increase the prevalence and load of bacteria in the airways. Among patients in a stable condition, larger bacterial loads in the airways were associated with more severe airflow restrictions and higher inhaled corticosteroid doses [169]. Additionally, it has also conclusively been shown that greater levels of interleukin (IL)-1, IL-10, and tumor necrosis factor (TNF)- in sputum were correlated with an increased bacterial load in the airways [170].

Moreover, individuals with stable COPD might be associated with a level of airway inflammation. Reducing the alveolar surface generated by emphysematous destruction has been suggested as a possible explanation for the relative growth of *Proteobacteria* and, to a limited extent, Actinobacteria. These bacteria are linked to the entry of neutrophils, eosinophils, and B cells in lung tissue [171]. Furthermore, a previous study by Segal et al. also found that the existence of oral microorganisms in the lung microbiome, which is enriched with *Prevotella* and *Veillonella* (supraglottic-characteristic bacteria), was associated with increased inflammation markers such as lymphocyte/neutrophil and highly linked to the increase of the Th17 lymphocyte [172,173].

Compared to *Prevotella*, *Haemophilus* has been demonstrated to have a nearly threefold inflammatory potential. *Haemophilus* species induce the expression of CD83, CD40, and CD86 in dendritic cells generated from human monocytes [174]. An intriguing finding is that the oral taxonomic enrichment of the lung microbiome increases microbiota metabolite concentrations and decreases alveolar macrophage TLR4 responses, with the latter leading to reduced infection clearance [172,174].

Several retrospective investigations utilizing culture-dependent methodologies have examined the incidence of *A. fumigatus* culture from lower airway specimens in patients with COPD [175,176]. In a large cohort of COPD patients hospitalized for severe exacerbation, *Aspergillus* was identified from 17% of patients’ sputum [177]. Running in parallel, the isolation of other pathogens, particularly *Pseudomonas aeruginosa*, was closely linked with an increased probability of the isolation of *Aspergillus* [160]. Tiew et al. (2021) provided the data of the COPD mycobiome in lower fungal diversity during acute exacerbation with two-year mortality. In this study, *Penicillium*, *Aspergillus*, and *Curvularia* were characterized as constituting a “high-risk” mycobiome. This group was found to show more exacerbation and greater symptoms compared to the group whose mycobiome was dominated by *Saccharomyces*. Additionally, this “high-risk” group tended to exhibit worse clinical outcomes and higher mortality; thus, early identification and clinical follow-up play a major role.

The investigation of the mycobiome also depends on the type of clinical samples obtained during study. Using oral wash and BAL from COPD patients, Cui et al. compared the topography of the mycobiome between these two types of respiratory samples [178]. The study found that *Ceriporia lacerata*, *Saccharomyces cerevisiae*, and *Penicillium brevicompactum* were significantly more abundant in BAL compared with oral wash [178]. A previous study reported that these three species might cause opportunistic lung infection [179,180,181]. Importantly, none of the control samples contained these species, implying the probability of these species existing as environmental contamination is implausible. The mycobiomes from patients with HIV infection and COPD were dominated with *Pneumocystis jirovecii*. These studies have demonstrated provocative associations between fungal and bacterial microbiota in the lung and COPD [21,22,178,182].

The remaining question, of how far the microbiome alteration in COPD might lead to *Aspergillus* colonization in COPD, has received considerable critical attention. The previous study investigated the usage of bronchodilators, and corticosteroid inhalation might be associated with altering the lung microbiome [121]. It has been hypothesized that steroid therapy will decrease some patients’ immunological response to the lung microbiota. As a result, the lung microbiome is likely to persist or expand [183].

The relevance of the mycobiome in the clinical progression of chronic respiratory diseases is clearly supported by the current findings. A recent study showed that the most common allergens detected in the houses of COPD patients were fungal allergens [184]. Moreover, the quantity of these allergens significantly correlated with the occurrence of COPD symptoms and decreased lung functions [184]. The variety of geographical aspects, the genetic defects of the immune system, and the crosstalk between organisms in the microbiome affect the clinical spectrum and the management of chronic respiratory diseases [60,184]. The management of chronic respiratory diseases might include the control of allergens related to the mycobiome aspect.

#### 4.1.2. Cystic Fibrosis (CF)

To date, a previous study has demonstrated that more than 50% of cystic fibrosis patients had *A. fumigatus* colonization, while the incidence of invasive aspergillosis among those patients was low [185]. As mentioned in the prior study, ABPA is likely the most clinical manifestation of *Aspergillus*-related disease in CF patients. A study conducted to observe bacterial microbiomes in CF patients has revealed that the colonization of *Pseudomonas* or *Streptococci* is predominant in CF patients in different states of diseases, either exacerbations or stable disease, respectively [186]. Both bacteria and fungi might be able to produce biofilms, and their interactions with pathogens potentially affect pathogenicity. Quorum sensing (QS) and cell-to-cell signaling are essential among the pathogen to ensure survival in the lungs of CF patients. Complex interactions between *Pseudomonas* and *Aspergillus* have been highlighted as being critical in the etiology of CF [111]. Intriguingly, the use of antibiotics against *Pseudomonas* spp. in CF patients was followed by a lowering number of aspergillosis in the sputum. These findings emphasize the critical role of *Pseudomonas* spp. and *Aspergillus* in the pulmonary milieu. Moreover, evidence has been reported that aspergillosis-metabolites produced are likely to affect the polymicrobial complex in CF patients [187].

It is believed that the mycobiome accounts for as little as 0.1 percent of the entire microbiome [30]. However, microbial environment perturbation induced by *Aspergillus* spp. and *Candida* spp. also might induce pulmonary diseases. Previous studies showed that mice deficient in dectin-1, a receptor in the fungal cell wall for β-glucans associated with innate immune response, had more potential to develop colitis due to an increase in the opportunistic fungi. They were also likely to generate an impaired immune response to those commensal fungi [188]. What is surprising is that the disruption induced by dectin-1, which also detects β-glucans from *A. fumigatus*, might alter the pulmonary immune response to fungi around the local environment. Likewise, genetic variations affecting dectin-1 have been previously linked to an increased risk of developing invasive aspergillosis (IA) in hematological patients [189,190]. Conclusively, studying dectin-1 might be a potential issue for providing additional insight into the structure and function of microbiota in genetic regulation perception.

Regarding microbiome interaction, *P. aeruginosa* colonies depict a different mechanism in inhibiting *A. fumigatus* among CF and non-CF patients [99]. They release a virulence factor called phenazines, which might disrupt the mitochondrial of *A. fumigatus* by producing reactive oxygen species (ROS) and reactive nitrogen species [100]. It has also been found that phenazines are associated with a better prognosis and more frequent pulmonary exacerbations [191].

Several studies have investigated the manner in which the *P. aeruginosa* QS system plays an essential role in interfering with the growth of *A. fumigatus* [131,132,192]. The QS system enables bacteria to enhance their pathogenicity, including migration and biofilm formation. Moreover, *P. aeruginosa* releases heat-soluble and diffusible molecules that mimic the QS molecule, leading to the decreased biofilm formation of *A. fumigatus* [131,132]. Furthermore, the QS system regulates other virulency molecules released by *P. aeruginosa*, including rhamnolipids and alkylhydroxyquinolones. Both molecules run their function; respectively, to disrupt the cell wall and biofilm integrity of *A. fumigatus* [102,103]. Furthermore, *P. aeruginosa* enhances its ability to inhibit the colonization of *A. fumigatus* through pyoverdine production. This molecule functions by capturing the iron from the milieu, inhibiting the biofilm formation of *A. fumigatus* [56,106].

Interestingly, amid the fungicidal mechanisms provided by *P. aeruginosa*, *A. fumigatus* seems able to thrive in CF patients. In fact, *A. fumigatus* might demonstrate the ability to reverse the antagonist activity of *P. aeruginosa* and may potentially restrict the growth of bacteria [131,193]. This is exemplified in the production of an antibacterial from *A. fumigatus*, well known as gliotoxin, which inhibits the biofilm formation of *P. aeruginosa* [105]. Additionally, the potential of *A. fumigatus* to generate its siderophores enables it to store iron, which is essential for survival, in the critical iron depletion environment. Moreover, to avoid antagonism by *P. aeruginosa*, *A. fumigatus* promotes the biotransformation of phenazine into a beneficial form such as phenazine-1-carboxylic acid (PCA), converting into 1-HP molecules leading to the self-generating of siderophore [194].

The most striking point regarding the interaction of *A. fumigatus* and *P. aeruginosa* is that these species provide a cooperative interaction model. The high prevalence of *A. fumigatus* colonies in CF patients after infection with *P. aeruginosa* suggests an exciting fact; that this bacteria colony may facilitate *A. fumigatus* growth [107,195]. Phenazine, known as one of the virulence factors released by *P. aeruginosa*, might enhance iron bioactivity at its low concentration, leading to *A. fumigatus* biofilm establishment. Moreover, the iron-chelating properties of phenazine also allowed *A. fumigatus* to adapt to the iron shortage, revealing its ability to adapt to the antagonist effect of *P. aeruginosa* [100,196]. On the other hand, *P. aeruginosa* may exhibit enhanced virulence due to phenotypic adaptations and genetic mutations accelerated by *A. fumigatus* [197].

#### 4.1.3. Chronic Pulmonary Aspergillosis (CPA)

CPA is likely characterized by infection with the *Aspergillus* genus, primarily associated with *A. fumigatus*, in individuals with pre-existing disease, especially chronic obstructive pulmonary diseases (COPD) or prior tuberculosis with no evident immunosuppression. This condition can be distinguished from invasive aspergillosis (IA) and allergic bronchopulmonary aspergillosis (ABPA), which are linked to immune dysfunction and hyperactivity (atopic), respectively. CPA is a severe lung disease within the pulmonary aspergillosis spectrum, and can progress to destroy parts of the lung [198,199].

Patients at a higher risk of CPA have a high variation in the pulmonary mycobiome [58,200,201]. The outcomes of the metagenomic study and real-time multiplex PCR show that patients at risk of CPA had a high *Aspergillus* prevalence [200,202]. It is vital to identify potential pathogenic fungi because HIV-positive patients are frequently misdiagnosed with pulmonary tuberculosis (PTB) when they may actually have CPA [200,203]. To avoid inaccurate diagnosis and unnecessary antibiotic use, means of identifying *Mycobacterium* spp. should also be tested [200].

A study by Zhao (2021) also shows that the interface between bacteria and fungi caused worsened clinical features. *Aspergillus* was the most common fungi detected in fungal and bacterial coinfection (43.8%) and fungal infection (36.6%) from the total of 119 patients [112]. Pulmonary cavity and immunocompromised status were identified as risk factors for fungal and bacterial co-infections [112]. There was a probability of CPA from the data mentioned since a proportion of the patients’ characteristics showed cavity lesions in CT imaging [112]. However, to have more reliable and conclusive data in the future, the analysis of different risk groups, a larger sample size, a more diversified range of patients, and healthy patients for comparison with the mycobiota in the study should be examined [200]. Increasing the sensitivity of the metagenomic approach by improving the collection of pure fungal DNA from the biological sample is also important [200].

### 4.2. Challenges in the Detection of the Lung Microbiome

Few studies have investigated lung microbiota systematically, owing to the challenge of describing the human lung environment using standard culture techniques relying on bacterial growth in bronchoalveolar lavage material [204,205]. This is because the lung has a lesser bacterial load than the other parts of the body, such as the gut and urinary tract. Additionally, there is debate regarding the possibility of microbial contamination from the lower respiratory tract with upper airways—this issue yields to the exclusion of the lung from the earlier study mapping the microbiome [204,206].

Several challenges also need to be taken into account regarding mycobiome sequencing. An essential consideration attributed to the contamination and DNA degradation with the fungal cell wall lysis might be a challenge in sample processing. Additionally, some bias emanating from the primer and amplification, target accuracy, and data reproducibility in the targeted amplicon sequencing process also contributed as a limitation in the sequencing of the mycobiome. In the stage of shotgun metagenomic sequencing, the issues attributed to the lesser fungal abundance compared to bacteria, difficulties of fungal detection due to enrichment of DNA host, and their high cost are also considered challenges to the sequencing.

Furthermore, some of the challenges might be associated with studying the respiratory tract microbiome. It is well known that there is an alteration in the microbiome distribution in chronic respiratory diseases; however, the frequency and distribution of these alterations are unknown. Additionally, regarding the sampling procedure, sputum might be the best sample to study the microbiome in the respiratory tract because it avoids invasive procedures. However, there is no denying that obtaining samples from the lower respiratory tract, like a peripheral bronchial tree and alveolar surfaces, likely provides reliable information. The contamination of the upper airway microbiome might be a confounding factor during the observational study of the lower airway microbiome. Furthermore, a systematic understanding of how complex microbiome–host interactions in different body sites contribute to respiratory diseases is still lacking. Analyzing the delicate crosstalk between host and microbiome in various human body sites may exhibit a remote impact from microbial communities and probably help to dissect the progression of chronic respiratory disease [207].

Another significant limitation in the microbiome study is related to the database analysis. Preliminary research must pick the relevant question and then select a suitable methodology from a selection of accessible bioinformatics tools [208]. It is also essential to evaluate the variation of the microbiome study, including sample heterogeneity, technical sampling, and the biases arising from DNA extraction sequencing [209]. An additional method, including metagenomic analysis, is necessary as 16S rRNA gene sequencing cannot reveal information about fungi and viruses in detail. Moreover, protocols must be standardized to conduct studies on the pulmonary microbiome involving sample collection, processing, and bioinformatics analysis [207].

Furthermore, regarding the condition of the respiratory system, several diseases, including CF and COPD, might reduce the diversity of the human microbiome in the respiratory tract. A similar pattern also can be seen in the gut microbiome, indicating that these phenomena might be linked to the operational taxonomic unit. A better understanding of the factors contributing to this loss of microbiomes, such as competition inter-species, antibiotic treatment, and host immunological responses, is essential [207].

## 5. Conclusions

Taken together, this review has elucidated that *Aspergillus* spp. induced a host immune response associated with a diverse range of bacteria–fungal interaction, comprised of cooperation and counter inhibition, in the respiratory tract, which influences microbiome colonization and the immunopathogenesis of CPA. However, this review was limited by the absence of a comparison between healthy and diseased lung models associated with microbiome colonization. Furthermore, there are several unexplored questions that should be investigated in the future as to whether the interaction between bacterial and fungal microbiomes in the normal respiratory tract reveals the same pattern in specific lung diseases. Additionally, due to several limitations in order to detect the lung microbiome, the combination of metagenomics and sequencing is urgently needed to explore the variety of the microbiome in the respiratory tract. Moreover, considerably more work will need to be carried out to determine the best sampling method for use in the lung to avoid contamination from the upper respiratory tract.

## Data Availability

Not applicable.
